# Treatment of arteriovenous fistula with aneurysm using the forearm branch of the cephalic vein as a candidate vessel: A case report

**DOI:** 10.1097/MD.0000000000032715

**Published:** 2023-01-20

**Authors:** Bo Wang, Ying Hu

**Affiliations:** a Division of Nephrology, West China Hospital of Sichuan University, Chengdu, China; b Department of Nephrology, People’s Hospital of Guizhou Province, Guiyang, China.

**Keywords:** aneurysm, Arteriovenous fistula, swollen hands, the excess cephalic vein branch

## Abstract

Arteriovenous fistula (AVF) is the preferred vascular access for hemodialysis patients due to its better patency rates and fewer complications than other access types. However, some complications related to AVF could not be ignored, including swollen hands and an aneurysm. Although a patient could not continue hemodialysis, it might threaten the patient’s life if the rupture of the aneurysm is not immediately treated by the surgeon. A report of using the cephalic vein branch to treat this complication is uncommon.

**Case presentation:**

Herein, we present a case of a 42-year-old man in whom an AVF with an aneurysm was successfully treated using the excess cephalic vein branch. This method is a simple and effective intervention for managing aneurysm-associated complications. Additionally, this approach helps maintain the benefits of autogenous access while conserving future dialysis sites.

**Conclusion:**

The surgery was effective and safe for this kind of complication with swollen hands and aneurysm. Using the excess cephalic vein branch could reconstruct the AVF.

## 1. Introduction

Arteriovenous fistulas (AVFs) provide the best vascular access for hemodialysis in patients with end-stage renal disease.^[1–3]^ However, some complications are associated with AVF, including bleeding, infection, venous thrombosis, swollen hands, and aneurysm. Among these complications, swollen hands, and aneurysm are common. Theoretically, swollen hands and aneurysms are caused by stenosis. In the treatment of these complications, the swollen hands can be managed by aneurysm and stenosis resection, but the AVF may be damaged, which might result in dialysis treatment difficulty in the next step.

## 2. Case presentation

**Figure 1.**
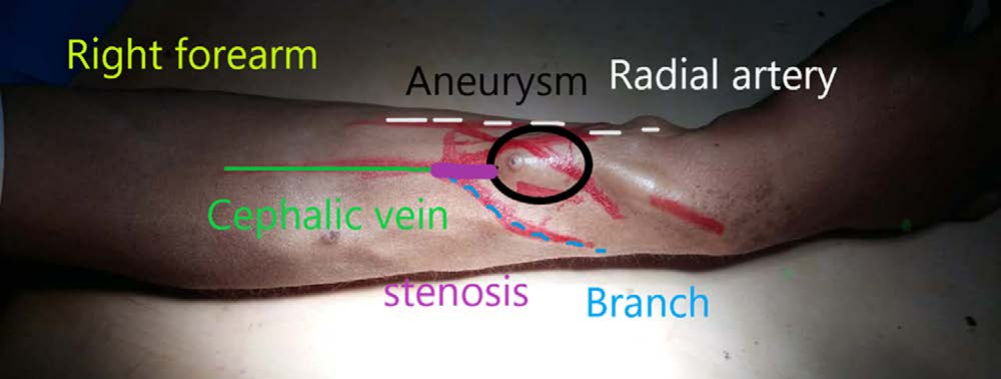
The right forearm before arteriovenous fistula surgery. Purple line: stenosis; Black line: aneurysm; green line: cephalic vein; blue line: the branch of the cephalic vein; white line: radial artery.

Herein, we report on a 42-year-old man with regular hemodialysis for 5 years. Two years before, the patient complained about swelling of the palm on the fistula side without pain. The surgeon suggested that the patient should have undergone vascular ultrasound to find the cause of his complaint. However, the patient did not cooperate well with the surgeon. In 10 days, the symptoms of swollen hands gradually became worse and painful. The pain was of grade 9 (1–10 score). The blood flow of the AVF did not meet the criteria of hemodialysis. Physical examination showed a 2.5-cm aneurysm within the anastomosis. The skin of the aneurysm was thin and red, indicating that the aneurysm might rupture (Fig. 1). Undoubtedly, the patient immediately needed surgery to avoid massive hemorrhage. Because the patient was young, the surgeon designed the method in which the AVF was reconstructed by the excess cephalic vein branch with radial artery while the aneurysm was removed to maintain the benefit of conserving the future dialysis site. The procedure was done under partial anesthesia after routine disinfection. An incision over the aneurysm was made longitudinally, and the arteries and veins were separated layer by layer from the aneurysm (Fig. 1).

**Figure 2.**
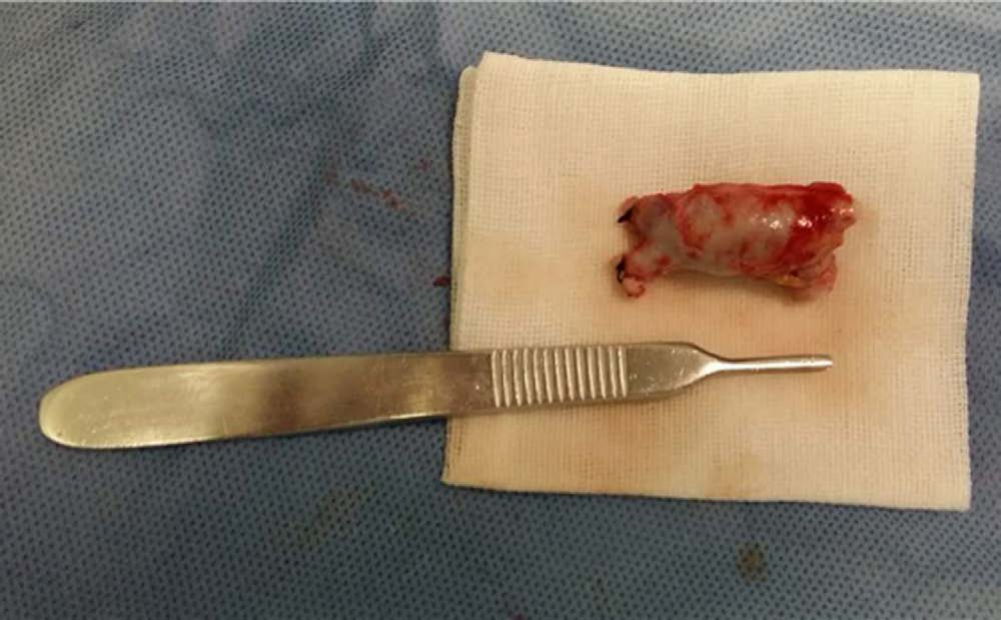
An aneurysm of about 2 cm was removed.

Proximal and distal control of the fistula was obtained using vascular clamps. Then the aneurysm was removed (Fig. 2). Additionally, the cause of the aneurysm was found: there was stenosis proximally to the aneurysm. Fortunately, the stenosis and aneurysm were located distally to the cephalic vein. The excess cephalic vein branch was explored. The diameter of the cephalic vein branch was 3.0 mm, which met the needs for an AVF suture. Lastly, the AVF was reconstructed by the excess cephalic vein branch. The patient felt a significant decrease in hand pain after the surgery. The patient was discharged the following day. Now, the patient is treated using this preserved AVF.

## 3. Discussion and conclusions

An aneurysm is common in AVF. The indication for the correction of an AVF aneurysm has been clearly established by the KDOQI guidelines.^[4]^ In our case, the patient was not regularly followed up but understood the importance of self-care after the AVF operation. The aneurysm grew rapidly, and there was an erosion of the overlying skin. The patient gradually felt that the pain in his hands worsened, and there was a risk of rupture and consequent fatal hemorrhage. The surgical removal of the aneurysm was necessary to protect the aneurysm from progressing. As a simple technique, the aneurysm may be managed by using many different techniques, either by surgery or endovascular procedures.^[5]^ One is to resect the adjacent skin, especially if it is damaged.^[6]^ The other is to use suturing with continuous polypropylene to form application of the “excess” wall as an acceptable method.^[7]^ However, these methods did not fit our patient. In our patient, his skin over the aneurysm was red, indicating a possible infection. The aneurysm was removed by surgery, and the reconstruction of the AVF was the tactic of treatment. However, the cephalic vein might have become short, and the utilization area would have been limited if the aneurysm and vein stenosis were directly removed. Fortunately, the excess cephalic vein branch of the patient was explored, which diameter met the criteria of the AVF reconstruction, suggesting that the first AVF surgery should retain the excess cephalic vein branch, especially since its diameter was more than 2.0 mm from the experience of this case. This may create a good way for the second AVF surgery.

In this report, the swollen hands and aneurysm were caused by vein stenosis proximal to the aneurysm. It was necessary to manage the stenosis to restore the function of the AVF. For outflow stenosis, surgical treatment and endovascular intervention are effective. In our case, the patient’s stenosis was very serious, which could not be repaired by these methods. Specifically, the vascular cavity of the stenotic segment disappeared. Therefore, the vein with the stenosis required surgical removal, suggesting that a follow-up after the first AVF was important to prevent complications. Some measures should have been taken for this condition. For patients, one measure is to maintain adequate self-care behavior to verify, maintain, and preserve the vascular access as functional.^[8]^ For example, the patient should be instructed not to allow venous punctures or blood pressure measurements on the arm where the access will be produced and protect the limb from injuries.^[9,10]^ The other measure is related to local hematoma formation when the patient should be taught to administer cold compresses during the first 24 hours of hematoma formation and warm the site after that period.^[11]^ Another caution is to not lie down over the arm with the AVF.^[12,13]^ A study in Iran with 110 hemodialysis patients has demonstrated that the aneurysm size of those patients that did not sleep on the fistula limb was significantly smaller than those that did. This fact emphasizes the association between the quality of self-care and the aggravation of complications, such as an aneurysm.^[13]^ For doctors, the surgical design of the first AVF is very important. In our case, the use of the patient’s cephalic vein branch was obvious, and its diameter met the criteria for the AVF. The surgeon retained the branch in the first operation, which provided the choice for the reconstruction of the AVF. Although the aneurysm developed, which resulted in the failure of hemodialysis treatment, the cephalic vein branch saved the AVF. This indicates that the cephalic vein branch that could meet the criteria for the AVF operation should be preserved when the surgeon first operates the AVF.

The follow-up was important after the first AVF surgery. Moreover, the patient should have immediately started self-care when the complication of AVF occurred. The surgeon’s design was essential in the first AVF operation, especially regarding the retainment of the cephalic vein branch. Finally, the excess cephalic vein branch saved the AVF after the swollen hands and aneurysm developed due to proximal vein stenosis of the aneurysm. The surgery was effective and safe.

## Acknowledgments

We acknowledge the support of the NHC Key Laboratory of Pulmonary Immunological Diseases (Guizhou Provincial People’s Hospital, Guiyang, Guizhou, China) and Yan Ran for her valuable help with the patient’s follow-up.

## Author contributions

Ying Hu conceived the idea of this article. Bo Wang collected data and wrote the manuscript. Ying Hu contributed to data collection and monitored the patient in the clinic. All authors read and approved the final manuscript.

Conceptualization: Ying Hu.

Data curation: Ying Hu.

Formal analysis: Ying Hu.

Software: Bo Wang.

Supervision: Ying Hu.

Writing—original draft: Bo Wang.

Writing—review and editing: Ying Hu.
